# Tom Pouce III, an Electronic White Cane for Blind People: Ability to Detect Obstacles and Mobility Performances

**DOI:** 10.3390/s21206854

**Published:** 2021-10-15

**Authors:** Aya Dernayka, Michel-Ange Amorim, Roger Leroux, Lucas Bogaert, René Farcy

**Affiliations:** 1 Laboratoire Aimé Cotton, Université Paris-Saclay, Centre National de la Recherche Scientifique, 91405 Orsay, France; aya.dernayka@universite-paris-saclay.fr (A.D.); roger.leroux1@orange.fr (R.L.); lucas.bogaert@universite-paris-saclay.fr (L.B.); 2 Complexité Innovation Activités Motrices et Sportives, Université Paris-Saclay, 91405 Orsay, France; michel-ange.amorim@universite-paris-saclay.fr; 3 Complexité Innovation Activités Motrices et Sportives, Université d’Orléans, 45067 Orléans, France

**Keywords:** electronic cane Tom Pouce III, white cane, avoiding obstacles, detecting obstacles

## Abstract

We present a protocol for evaluating the efficiency of an electronic white cane for improving the mobility of blind people. The electronic cane used during the test is the Tom Pouce III, made of LIDAR sensors (light detection and ranging) with tactile feedback. The protocol comprises two parts. The first part, the “detection test”, evaluates the efficiency of the sensors in the Tom Pouce III for detecting the obstacles found in everyday life (thin and large poles, apertures) under different environmental conditions (darkness, sun light, rain). The second part of the test, the “mobility test”, compares the ability of blind participants to cross a 25 m path by avoiding obstacles with the simple white cane and the electronic cane. The 12 blind participants had between 2 and 20 years of experience of everyday usage of Tom Pouce devices. The results show a significant improvement in the capacity to avoid obstacles with the electronic cane relative to the simple white cane, and there was no speed difference. There was no correlation between the results and the years of experience of the users.

## 1. Introduction

For more than 80 years, the white cane and the guide dog have been the only assistive devices used in everyday life by blind people to assist walking independently. The white cane only anticipates obstacles by touching them, a few tens of centimeters before the point at which the body would contact them. The head of the user is not protected by the cane. For these reasons, several works have been carried out to develop an electronic cane that allows blind people to avoid obstacles with greater anticipation and safety.

We name below some examples of the devices found in the literature with different kinds of sensors:

- ultrasonic devices—Sonic Torch [1], Nottingham Obstacle Detector [2], Mowat sensor [3,4], and UltracaneTM [5,6,7];
- camera devices—Researchers tried to use a smartphone camera to scan the area and then send alerts via the earphones, such as in the vOICe [8], or used image processing to detect objects, such as in Sandness [9];
- lidars—Laser Cane (1992), Teletact I (1998) [10], Tom Pouce I (2000) [11], Teletact II (2004), Tom Pouce II (2006) [11], Tom Pouce III (2012) [12], Eye Cane (2014) [13]. See also studies by Gomez and Sandnes (2012) [14] and Pallejà et al. (2010) [15] and an assistive device based on millimeter wave radar technology (2018) [16].

This list is not exhaustive but illustrates the diversity of the sensors used in the electronic canes. For most devices, tests have not been carried out to check the ability of the device to detect objects. There is also a lack of tests showing the ability of visually impaired people to avoid obstacles in real situations with electronic devices. For example, tests have used blind participants with no experience of the electronic device or naive blindfolded sighted participants [8,9,10,11,12,13,14,15,16,17,18,19]; other tests have been carried out that have no correlation with real life, such as following a lighted line on the floor in a dark room [20], etc.

Effective tests are very important because different technologies do not detect obstacles with the same efficiency given their different operative processes, such as use of a camera (passive light), lidar (active light) or ultrasonic technology (ultrasounds). There are many parameters that may affect performance. More precisely, the ability of a sensor to detect an obstacle (or a path) may depend on many physical factors, such as the roughness of the surface, width, color, inclination, luminosity, humidity, etc. For example, ultrasonic sensors can consider rain as an obstacle, and their sensibility is dependent on the shape of the obstacle. Passive camera devices are affected by luminosity. Lidars can be affected by high luminosity, and many sensors do not detect thin pikes. The perfect sensor does not exist; therefore, an evaluation of the performance of the sensors, under real conditions of use, in various situations representative of everyday life for blind people, is important. The literature on the performances and limits of the sensors used in electronic canes is sparse. Only a few references mention the limitations of the sensors, such as [2,7,11].

Another important point is the transfer of information by the sensors to the blind person. To evaluate that point, we need to test the ability of blind people to find their path in cluttered environments, as in a real-life situation. For most systems, we did not find reports of reproducible tests mimicking real-life situations. 

Here, we suggest a protocol that is as close as possible to the everyday life of the users in order to assess the utility of an electronic white cane. The test comprises two parts:

- The first part of the test evaluates the detectivity of the electronic cane in a real dynamic situation, i.e., in a situation taking into account the movement of the user.
- The second part of the tests evaluates the ability of the blind user to navigate a cluttered environment without collisions.

The tests were performed with the electronic cane “Tom Pouce III”, which is produced by VISIOPTRONIC (not involved in our study), who are owned by the VISIO foundation. Currently, there are 950 users in France, who receive two weeks of training and an annual evaluation to teach them how to properly use the cane.

## 2. Materials and Methods

### 2.1. Participants

The first part of the test involved three sighted trainees.

The second part involved blind people from category V (according to the World Health Organization classification) using the Tom Pouce III electronic cane in their daily lives for at least two years. All the participants were blindfolded with “Goal fix Eclipse total” blackout eyeshades.

All the blind participants received 30 h of training to use the Tom Pouce III. The course was given by a mobility specialist. One of the main focuses of this course was to make sure the sweeping of the cane was synchronized with the user’s steps, and that the width of the sweep was large enough to protect them. All participants were recruited on a voluntary basis (age range, 31–64) after contacting their rehabilitation center. We asked for a list of users who would agree to participate in a mobility behavioral study using their electronic cane. They were selected randomly on the basis of two criteria: they should live in Paris or in the suburbs of Paris and should have at least two years of daily experience with the cane. All documents regarding the study were sent by mail to the participants and were further explained by phone if needed. The protocol was accepted by the Université Paris-Saclay Polethis’ ethical committee under the reference CER-Paris-Saclay-2019-038. All the participants received a coupon for their involvement.

### 2.2. Description of the Tom Pouce III

**Figure 1:**
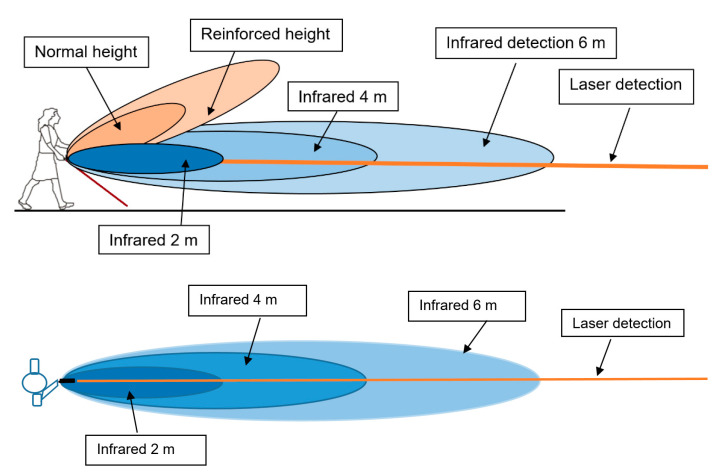
Illustration of Tom Pouce III detection ranges. **Top panel**: lateral view of the protection area of the Tom Pouce. **Bottom panel**: top view of the protection area.

Two types of sensors are implemented in Tom Pouce III: an infrared LED sensor and a red laser sensor. The laser detector is a triangulation laser telemeter and is as thin as the laser beam. The infrared detector has a larger field. The field of infrared detection is determined by a cylindrical revolution around the laser’s axis for frontal detection. Figure 1 represents the protection area and ranges of the different sensors.

The red laser detector and the infrared LED sensors work independently and are complementary. A simple “OR” logical function is used to fuse the two feedbacks (if “obstacle” for one sensor and “no obstacle” for the other, we conclude “obstacle”). 

There are two limitations related to the laser sensor. Firstly, the laser beam is very thin, and small apertures cannot be detected. However, infrared LED sensors cannot detect small apertures, and they compensate for this defect via the OR function. The second limitation of the triangulating laser telemeter is its inability to detect between 0 and 12 m. To ensure good detectivity at greater distances, detectivity at shorter distances must be sacrificed. As such, the minimum distance of detection of the laser sensor is 1 m, and detection becomes efficient above 1.2 m. On the other hand, the infrared LED sensor can detect from 0 m.

The main limitation of the infrared LED sensor is the impossibility of detecting narrow dark posts. In this case, the amount of light retrodiffused to the sensor by the post is not sufficient, because the post is narrow and black and occupies only a very small part of the illuminated area. On the other hand, a thin laser beam can concentrate all its energy on the narrow post, and thus detect it.

The user can choose between 2, 4 and 6 m detection ranges. Here, the laser and infrared detection are both limited to the same value—2, 4 or 6 m. If the user chooses the 12 m detection range, the laser can achieve 12 m detection, but the infrared detection range remains limited to 6 m. 

An alert is provided through vibrations sent to the palm: a smooth vibration for the first level of alert (less than 4, 6 or 12 m, depending on the range chosen by the user), and a strong intermittent vibration when the distance is under 2 m.

### 2.3. The “Detection Test”

To describe the behavior of the sensor, we considered four categories of obstacles under three different conditions:

1. large white pole—including people with light clothes, electrical poles, trees;
2. large dark pole—including people with dark clothes, telephone poles;
3. thin dark pole—including black anti-parking poles, thin tree branches without leaves; and
4. thin white pole—including white anti-parking poles.

The three different conditions were:

1. total darkness;
2. medium luminosity with rain; and
3. high luminosity.

We also measured the ability to detect different door apertures (25, 50, 75, 100 and 125 cm) at many distances (1, 2, 4, 6 and 12 m) by sweeping the tip of the cane. 

To describe the movement of the cane while walking, we adopted the recommendations of the mobility course [21]. While walking, the blind person coordinates their steps with the tip of the cane. For example, when the person takes a step, the cane sweeps from left to right or from right to left. The width of the sweep is equal to the shoulders’ width.

The rotation speed of the cane while sweeping is crucial for aperture detection. We considered several standard parameters for a 1.70-m-tall person with a 50 cm shoulder width. Given a 65 cm step length and a 0.6 s step duration (step cadence 1/0.6 = 1.66 Hz), the walking speed would be 0.65 m/0.6 s = 1.08 m/s or 3.7 km/h. The recommended length of the cane under these conditions is 1.3 m. Here, the distance from the tip of the cane to the front foot of the user, measured along the floor, would be about one meter, and the angular width of the sweep would be about half a radian. The average rotation speed of the cane would be 0.5 rad × 1.66 Hz = 0.8 rad/s. In addition to these normal walking parameters, we also considered slower walkers. In sum, the walking conditions were as follows:

○ 3.7 km/h (1.08 m/s) for normal walking speed with a sweeping rate of 1.66 Hz, corresponding to a 0.8 rad/s cane rotation speed;
○ 1.85 km/h (0.54 m/s) for reduced walking speed, with a 0.88 Hz sweeping rate and a 0.4 rad/s cane rotation speed; and
○ 0.925 km/h (0.254 m/s) hesitating walking speed, with a 0.44 Hz sweeping rate and a 0.2 rad/s cane rotation speed.

**Figure 2:**
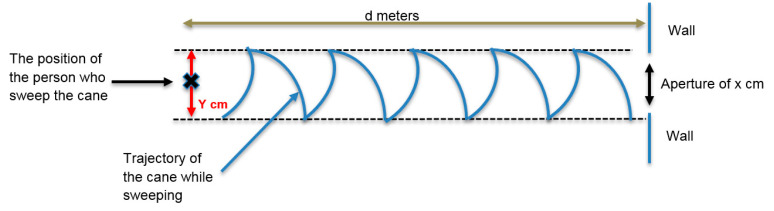
A descriptive illustration of the movement of the tip of the cane while walking.

Figure 2 represents the trajectory of the rolling tip of the cane for a person walking toward a wall. Here, *d* is the distance of the person from the wall, and *Y* is the width of the cane tip’s sweep.

**Figure 3:**
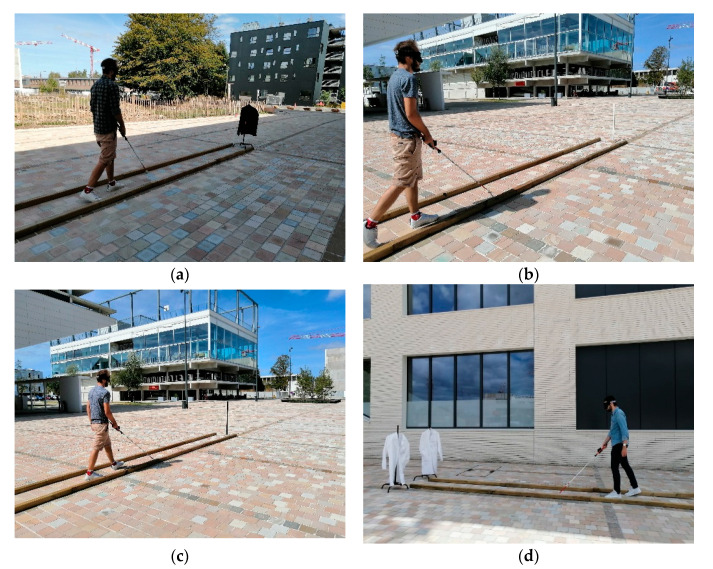
A sighted blindfolded participant sweeping the cane between the two bars at a given cadence to detect (**a**) a large dark obstacle under intermediate luminosity conditions; (**b**) a white thin post in full sunlight; (**c**) a dark thin post in full sunlight and (**d**) an aperture.

We used the following process to check whether or not the cane can be used to detect under standard walk conditions at different distances. We fixed the distance *d* (6 m for example), while the width of the cane tip’s sweep (50 cm for example) was limited by parallel bars on the floor (Figure 3). We used a metronome to determine the cane’s sweep at a set step cadence (1.66 Hz for example). For a thin obstacle, if the user felt a vibration in the direction of the obstacle at the given sweeping rate, the obstacle was considered as detected by the sensors. For an aperture, if the user felt no vibration in the direction of the aperture at the given sweeping rate, it was considered detected.

### 2.4. The “Mobility Test”

**Figure 4:**
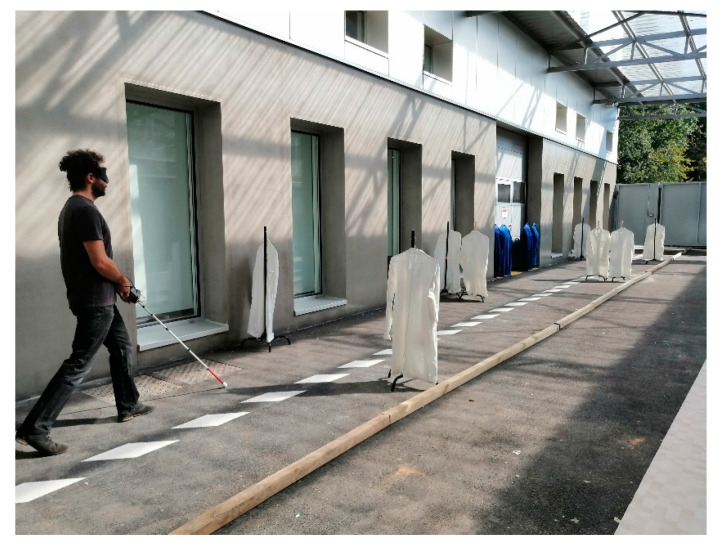
We see a participant walking down a 25 m long and 2.4 m wide path; 7 cm wooden square bars were positioned on the left side of the path, and a wall was on the right side. Obstacles made of lab coats on coat racks were placed along the path.

The test path was 25 m long and 2.4 m wide, with a wall on one side. On the other side, 7 cm square wooden bars limited the width of the path. This configuration was intended to reproduce a sidewalk. The obstacles were coat racks, 60 cm wide and 1.2 m high, used to simulate the presence of a person or a trash can on the path (Figure 4).

The instructions given to the participants were as follows:

- The path must be navigated without any contact with the obstacles or the wall. Every contact with an obstacle (with the cane or the body) will affect the score of the test.
- The first trial will include only one obstacle.
- If the path is walked without contact within 45 s, the next path will contain one more obstacle. The position of each obstacle changes in each attempt.
- In the case of a contact, the participant must stop and attempt the path again with the same number of obstacles. The position of each obstacle is changed in each path.
- The mobility test ends at the fifth contact.

The first test was randomly assigned either the cane or the Tom Pouce III. We defined the score of the mobility test as follows: *N* means that the person has succeeded in traversing a path containing “*N*” obstacle(s) without contact before the fifth contact. Possible scores were between zero and eight.

If the user reached a score of “8” with less than five contacts in total, he/she was asked to traverse a path with eight obstacles as fast as possible. The trial ended after the fifth contact. Each walk was recorded via a camera held by an investigator following the participant. The other investigator changed the position of the obstacles for the next attempt.

## 3. Results

In this section, we present the results obtained in the “detection test” and the “mobility test”.

### 3.1. Results for the “Detection Test”

In the Table 1 and Table 2 below, we used the same measurement method as explained above (see Figure 3). The “lux” is a unit of measurement of diurnal light. Specifically, 0 lux is full darkness, and 100,000 lux is the maximal illumination possible on Earth. The objective was to verify whether the device correctly detected obstacles under high and low sunlight conditions. Table 1 shows the results for a large (dark or white) or thin white obstacle and Table 2 for a thin dark obstacle.

These obstacles were easily detectable by active optical methods under any environmental conditions. The Tom Pouce III’s vibration is of minimal duration, so if the user wants to determine between a 4 and a 50 cm obstacle, he/she has to perform a slow sweeping movement of the cane. At normal walking speed, the deviation required to avoid a 4 cm object can be the same as that for a 50 cm object; distinguishing between them is not so important. 

**Figure 5:**
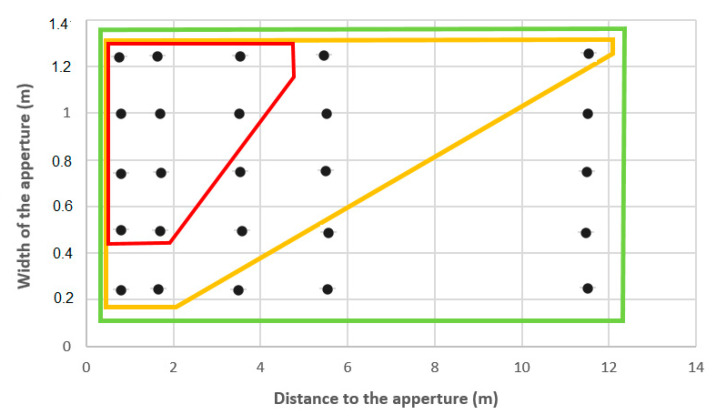
Detection performance for apertures, with black points representing points of measurement from different distances. The red parallelepiped contains points indicating apertures detected at 0.8 rad/s; those inside the yellow parallelepiped indicate apertures detected at 0.4 rad/s and the green parallelepiped indicates full aperture detection at 0.2 rad/s.

Figure 5 shows the detection performance for several door apertures (25, 50, 75, 100 and 125 cm), as a function of the distance to the aperture (0.8, 1.7, 3.5, 5.5, and 11.5 m) and the sweeping speed of the cane (0.8, 0.4, and 0.2 rad/s).

Let us consider, for example, the point in the top right-hand corner, which corresponds to an aperture of 1.25 m seen at 11.5 m. If the sweeping speed of the cane was 0.2 or 0.4 rad/s, the aperture was detected. However, if the sweeping speed was 0.8 rad/s, the 1.25 m aperture was not detected.

### 3.2. Results for the “Mobility Test” 

**Figure 6:**
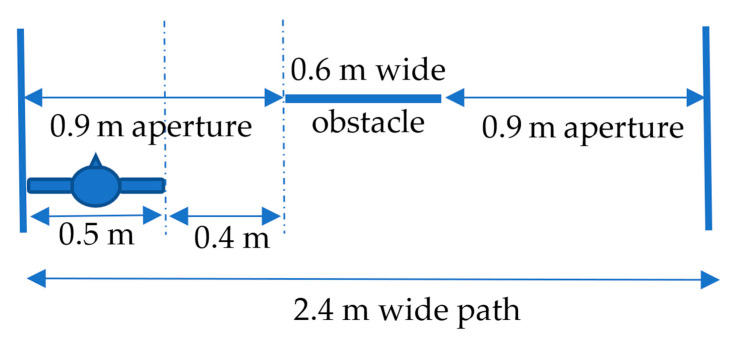
Schematic top view of the experimental setup illustrating the parameters used for computing the probability of passing the apertures without collisions by chance.

A performance score was computed to account for the fact that a participant could walk through an aperture without touching obstacles by chance (see Appendix A for details). Indeed, when there are only one or two obstacles (60 cm width) in a 2.4-m-wide path, there is a good probability that someone can walk it without touching an obstacle, only by chance. Therefore, we evaluated the probability of achieving a given score via a random trajectory (see Table 3). To calculate the probability associated with a random trajectory, we estimated the probability of passing the obstacles by chance, considering the width of the path, the width of the person (typically 50 cm) and the widths and positions of the obstacles. The length of the free space available to navigate the path without contact divided by the scope of the possible positions of the person on the path gives the probability of passing an obstacle by chance. For example, the probability of passing an obstacle in the middle of the path by chance is less than that for the same obstacle on the side of the path. When the obstacle is larger, the chance is lower. The greater the number of obstacles, the lower the chance. We illustrate in Figure 6 the parameters on which the calculation was based. The path was 2.4 m wide, and we considered an obstacle 60 cm wide in the center of the path. The left and right apertures were 90 cm wide. The person was 50 cm wide, so the possibility of entering the left path without collision by chance was (90–50 cm)/(240–50 cm), i.e., 0.21. For both apertures, the probability was then 0.42—a probability of one in three. We had to take into account the position and the width of each obstacle, and the different combinations, in order to calculate the probability of each score. 

**Figure 7:**
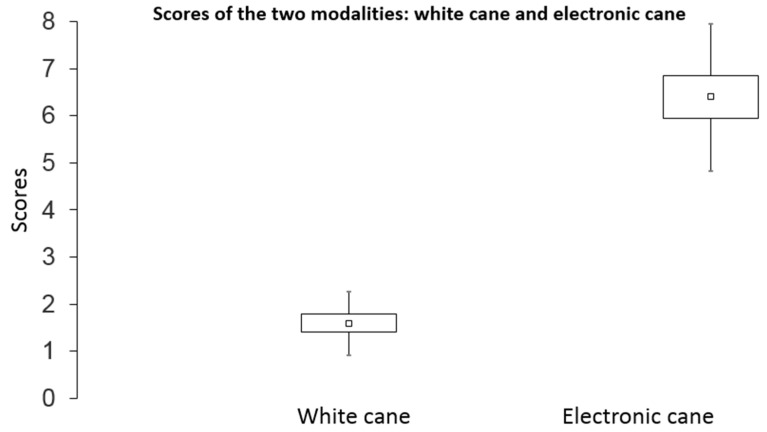
Effect of using the simple white cane and Tom Pouce III on the mobility score (±SD = error bar; ±SE = square).

The mean scores of the blind participants in the mobility test are provided in Figure 7. For a simple white cane, the average score was 1.6 (standard deviation = 0.67; standard error = 0.19), whereas with the Tom Pouce III, the average score was 6.4 (SD = 1.56; SE = 0.45).

The most likely (*p* = 0.43) score obtained with a random trajectory was “2” (see Table 3) with an average score given by chance of 2.1 (from the products of scores and probabilities). The results show that the obstacle avoidance score when using the white cane (*M* = 1.6) did not differ from chance (0.227 < *p* < 0.429), whereas the obstacle avoidance score when using the Tom Pouce III (*M* = 6.5, *p* = 0.000027) was greater than chance. 

**Figure 8:**
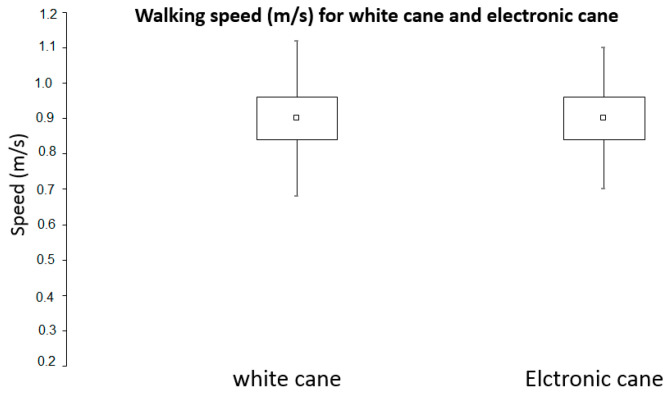
Effects of the simple white cane and Tom Pouce III on mean walking speed.

The Student paired *t* tests for comparing mean sores between the white cane and the Tom Pouce III (Figure 7) indicated significantly greater scores when using the Tom Pouce III (*t*(11) = 10.12, *p* = 0.00000066). The mean walking speed of participants in m/s is reported in Figure 8. The average speed of the 12 participants was 0.9 m/s when using the simple cane, and the speeds when using the electronic cane (with, respectively, SD = 0.22 and 0.2, and SE = 0.06 and 0.06) were very similar under both modalities. There was no significant difference in mean walking speed as a function of the cane, (*t*(11) = 1.32, *p* = 0.21), suggesting that the difference in score cannot be attributed to a difference in walking speed. In other words, there was no speed–accuracy tradeoff in our data.

Participants 4, 5, 7, 9 and 12 succeeded in walking the path with eight obstacles without contact. As such, they were asked to navigate it as fast as possible until failing all the trials and reaching the fifth contact. Accordingly, the greatest speeds reached for the trial with eight obstacles without contact were 2.5 km/h for participant 4, 5 km/h for participant 5 (participant 5 reached a score of 8 without any contact), 5.3 km/h for participant 7, 5 km/h for participant 9 and 5.6 km/h for participant 12.

Finally, we did not find any correlation in each participant between year of birth, years of blindness, years of experience with the white cane and with the electronic Tom Pouce III cane and the mobility scores as we can see in Table 4.

## 4. Discussion

### 4.1. Detection of Obstacles

Different weather condition raised no problems with our obstacles. However, it must be remembered that it took 30 years of development and improvement to reach that goal. Thin dark poles are difficult to detect by active optical methods. We explain in Section 2.2 that the infrared LED sensor does not detect narrow dark poles and that the red laser sensor only works for distances above 1 m. Therefore, it is to be expected that dark narrow poles will not be detected at 0.8 m. Under the normal use conditions of the device, dark poles have to be anticipated by the user at a few meters. When the user is facing an unanticipated dark pole at less than one meter, the electronic white cane will vibrate to indicate the pole. At long distances, on a sunny day and at a high walking speed, the utility of the laser sensor is limited. In this case, a narrow dark pole will be the most difficult obstacle to detect.

### 4.2. Detection of Apertures

It is easier to see small apertures at large distances with a reduced sweeping speed of the cane. Because a 25 cm aperture is too small for a human to go through without difficulty, it is a functional advantage to not detect such small apertures at great distances so that a larger path can be selected instead. This occurs at a normal walking speed. 

However, we found that with a 50 cm sweep of the cane at a speed of 0.8 rad/s together with a 3.8 km/h normal walking speed (see the red parallelepiped in Figure 5), the ability to detect apertures was poor. Accordingly, a 0.75 m aperture corresponding to a door will not be detected at 3.5 m. Nevertheless, electronic cane users get around this difficulty. In fact, if the tip of the cane sweeps the width of the shoulders (50 cm wide, 1 m ahead), the detection area with a 4 m range is four times greater, given a 2 m sweep; at 12 m, the sweep would be 6 m. However, considering such information may impede the fluidity of walking. Therefore, all users of an electronic cane should reduce the sweep of the cane to the width of the feet in order to detect positive and negative height differences in relation to the front foot. The width of the feet is about half the width of the shoulders. As a consequence, electronic cane users should reduce the standard sweep by half (about 25 cm). Shoulder protection will be given by the sensors and feet protection by the tip of the cane. For example, at 3.8 km/h, with a 25 cm large sweep, the sweeping speed is divided by two, i.e., 0.4 rad/s. The corresponding aperture detection at a normal walking speed is illustrated by the yellow parallelepiped in Figure 5. Apertures that were too small for the body (e.g., 0.25 m wide) could not be detected two meters away, but a 75 cm door aperture could be correctly detected at 6 m. 

### 4.3. The Mobility Test

Although all the participants were blind (category V), they were blindfolded with a “Goalfix”, a blackout eye mask used for official Paralympic competitions. They confirmed that it did not disturb their performance. With a simple cane, the mean score was 1.6, which is close to random walking performance. In contrast, with a Tom Pouce III, the mean score of participants was 6.4. The probability of getting this score by chance is 3 × 10^−5^, that is, 1 in 33,000. This suggests that the Tom Pouce III allows the distant perception of obstacles, providing an advantage (with respect to the white cane) in obstacle anticipation and walking fluidity. The mean walking speeds with the cane and Tom Pouce III were not different, suggesting that the Tom Pouce III did not induce a cognitive load that would affect walking speed. Beginners would not be able to carry out these tests at a normal speed, since several months of experience are required before reducing the cognitive load when using the device. Note that the probability of reaching the highest score without contact by chance is one in one billion, which participant 5 achieved. 

Our participants had used the Tom Pouce III in everyday actives for at least 2 years and up to 20 years (for previous generations), and their performances were stable while using the device. Their cane sweep widths were clearly less than their shoulder widths, but a little greater than the widths of their feet. As such, at a normal walking speed (3.7 km/h), the perception of apertures by trained electronic cane users is accurate. The spontaneous reduction in the width of the sweep by users was at first a source of stress for mobility specialists. In actuality, the feet are protected in the vertical plane, and the lateral protection derived is greater than that given by the white cane, which is 1 m, when the laser beam is projected up to 6 m ahead. Even if the amplitude of the movement of the tip of the cane is reduced by half, the lateral perception is better with the electronic cane.

One limitation of our tests may reside in the fact that our long-term electronic cane users may have lost part of their ability to use a simple white cane. For this kind of test, passive or active echolocation [22,23] may be the only possibility for avoiding obstacles (excluding chance). Indeed, the obstacles we used were made of clothes, were less than 1.2 m high and difficult to detect with “echolocation” at a normal walking speed. Some of our users who have been blind since birth have very good echolocation abilities, but none of them could achieve a better score than 3 with the white cane. None of the users of the simple cane made contact with the wall, because this obstacle is easily detectable by passive echolocation, thus indicating they have not lost this ability.

We plan to repeat the tests in order to investigate intra-individual reproducibility after a few months. In addition, it would be interesting to assess beginners using the Tom Pouce III, to see how quickly one can progress from a score of 2 to 7. Finally, even when achieving a score of 6, we noticed defects of handling the device among the participants. It would be useful to examine whether some reeducation may help to correct these defects, and whether this could affect their score. 

## 5. Conclusions

To conclude, in further investigations, we will focus on the detection of particular obstacles such as sloped glass, the capacity for lateral protection in the case of small dark anti-parking poles and the effects of special weather conditions, such as fog, snow, and so on. In mobility tests, we plan to assess the endurance of users traversing a very long cluttered path, in order to quantify the cognitive load induced by the use of the electronic cane. The difference in performance between the simple white cane and a white cane with Tom Pouce III is significant. However, test–retest experiments will be useful to measure the reliability of the mobility scores. Finally, we should emphasize that our protocol does not require specific or expensive materials and is very easy to reproduce with another device or a future generation of the same device, in order to compare their relative efficiency.

## Figures and Tables

**Table 1:** This table shows the results of detection for a large dark obstacle at different distances, sweeping speeds and illuminations (D means detected, and ND means not detected). The results were the same for large and thin white obstacles, so we summarize the three cases in the same table.

Large Dark or White Obstacle (Length: 1.2 m; Width: 50 cm), Thin White Obstacle (Length: 1.5 m; Width: 4 cm)												
Lux	0 lux (full darkness)			0.01–5000 lux (case of rain)			5000–50,000 lux			>50,000 lux		
Sweeping speed (rad/s)	0.2	0.4	0.8	0.2	0.4	0.8	0.2	0.4	0.8	0.2	0.4	0.8
Distance (m)												
0.8 m	D	D	D	D	D	D	D	D	D	D	D	D
1.7 m	D	D	D	D	D	D	D	D	D	D	D	D
3.5 m	D	D	D	D	D	D	D	D	D	D	D	D
5.5 m	D	D	D	D	D	D	D	D	D	D	D	D
11.5 m	D	D	D	D	D	D	D	D	D	D	D	D

**Table 2:** This table shows the results of detection for a thin dark obstacle at different distances, sweeping speeds and illuminations (D means detected, and ND means not detected).

Thin Dark Obstacle Detection (Length: 1.5 m; Width: 4 cm)												
Lux	0 lux (full darkness)			0.01–5000 lux (case of rain)			5000–50,000 lux			>50,000 lux		
Sweeping speed (rad/s)	0.2	0.4	0.8	0.2	0.4	0.8	0.2	0.4	0.8	0.2	0.4	0.8
Distance (m)												
0.8 m	ND	ND	ND	ND	ND	ND	ND	ND	ND	ND	ND	ND
1.7 m	D	D	D	D	D	D	D	D	D	D	D	D
3.5 m	D	D	D	D	D	D	D	D	D	D	D	D
5.5 m	D	D	D	D	D	D	D	D	D	D	D	D
11.5 m	D	D	D	D	D	D	D	D	D	D	D	ND

**Table 3:** Probability of a given score with a random trajectory.

Score	0	1	2	3	4	5	6	7	8
Probability of getting the score at random	0.02	0.23	0.43	0.26	6.1 × 10^−2^	7.2 × 10^−3^	4.3 × 10^−4^	1.5 × 10^−5^	1.8 × 10^−7^

**Table 4:** This table shows the date of birth of each user, the year of full blindness, the period of use of the white cane and the period of use of the Tom Pouce I, II or III.

Participant	Birth Date	Full Blindness Date	Period of Use of the Simple White Cane	Period of Use of Tom Pouce I, II or III
1	1982	2012	1990–2009	2009–2021
2	1979	1983	1990–2015	2015–2021
3	1983	1984	1995–2001	2002–2021
4	1990	1990	2000–2015	2015–2021
5	1987	1997	1997–2014	2015–2021
6	1972	1985	1990–2010	2010–2021
7	1976	1988	1986–2001	2001–2021
8	1963	1963	1970–2010	2010–2021
9	1958	1980	1975–2013	2013–2021
10	1965	2006	1978–2019	2019–2021
11	1963	2016	2015–2016	2017–2021
12	1972	1980	1984–1994	2017–2021

## Data Availability

Data available on request.

## Appendix A

MATLAB code used to calculate the probability by chance of each score for the mobility test

% Score 0: five consecutive collisions with a single obstacle on the way.

T1 = 0.421; % probability of avoiding a simple obstacle without collision,

E1 = 1 − T1; % probability of collision with a single obstacle on the way,

Score 0 = A5C1N(E1); % probability of score 0, i.e., five consecutive collisions with one obstacle on the way.

% Score 1: finalized with a collision with two obstacles on the way after four collisions distributed with attempts including one or two obstacles on the way.

T2 = 0.421 × 0.421;

E2 = 1 − T2;

Score 1 = T1 × E2 × A4C2N(E1,E2); % probability of reaching score 1;

% T1 probability of avoiding a single obstacle, E2 probability of the fifth collision with two obstacles, A4C2N(E1,E2) probability of four collisions distributed with attempts including one or two obstacles on the way.

% Score 2: finalized with a collision with three obstacles on the way after four collisions distributed with attempts including one or two or three obstacles on the way.

T3 = 0.421 × 0.421 × 0.421;

E3 = 1 − T3;

Score 2 = T1 × T2 × E3 × A4C3N(E1,E2,E3);

% T1T2 of avoiding one obstacle on the way and then two obstacles on the way, E3 probability of the fifth collision with three obstacles, probability of four collisions distributed with attempts including one, two or three obstacles on the way

% Score 3: finalized with a collision with four obstacles on the way after four collisions distributed with attempts including one, two, three or four obstacles on the way.

T4 = 0.421 × 0.421 × 0.421 × 0.421;

E4 = 1 − T4;

Score 3 = T1 × T2 × T3 × E4 × A4C4N(E1,E2,E3,E4).

% Score 4: finalized with a collision with five obstacles on the way after four collisions distributed with attempts including one, two, three, four or five obstacles on the way.

T5 = 0.421 × 0.421 × 0.421 × 0.25; 0.25 probability of avoiding by chance an obstacle of double width;

E5 = 1 − T5;

Score 4 = T1 × T2 × T3 × T4 × E5 × A4C5N(E1,E2,E3,E4,E5).

% Score 5: finalized with a collision with six obstacles on the way after four collisions distributed with attempts including one, two, three, four, five or six obstacles on the way.

T6 = 0.421 × 0.421 × 0.25 × 0.25;

E6 = 1 − T6;

Score 5 = T1 × T2 × T3 × T4 × T5 × E6 × A4C6N(E1,E2,E3,E4,E5,E6).

% Score 6

T7 = 0.421 × 0.25 × 0.25 × 0.25;

E7 = 1 − T7;

Score 6 = T1 × T2 × T3 × T4 × T5 × T6 × E7 × A4C7N(E1,E2,E3,E4,E5,E6,E7).

% Score 7

T8 = 0.25 × 0.25 × 0.25 × 0.25;

E8 = 1 − T8;

Score 7 = T1 × T2 × T3 × T4 × T5 × T6 × T7 × E8 × A4C8N(E1,E2,E3,E4,E5,E6,E7,E8).

% Score 8

Score 8 = T1 × T2 × T3 × T4 × T5 × T6 × T7 × T8 × A4C8N(E1,E2,E3,E4,E5,E6,E7,E8).

Score 8P = T1 × T2 × T3 × T4 × T5 × T6 × T7 × T8 % score eight without any collisions.

Functions:

function[res] = A5C1N(a)

res = a^5^;

end

function[res] = A4C2N(a,b)

res = a^4^ + a^3^ × b + a^2^ × b^2^ + a × b^3^ + b^4^;

% sum of the probabilities of four collisions spread over paths including one to two obstacles

end

function[res] = A4C3N(a,b,c)

res1 = a^4^ + a^3^ × (b + c) + a^2^ × (b^2^ + b × c + c^2^) + a × (b^3^ + b^2^ × c + b × c^2^ + c^3^);

res = res1 + A4C2N(b,c)

% sum of the probabilities of four collisions spread over paths including one to three obstacles

end

function[res] = A4C4N(a,b,c,d)

res1 = a^4^ + a^3^ × (b + c + d) + a^2^ × (b^2^ + b × (c + d) + c^2^ + c × d + d^2^);

res2 = a × (b^3^ + b^2^ × (c + d) + b × (c^2^ + d^2^ + c × d) + c^3^ + c^2^ × d + c × d^2^ + d^3^)

res = res2 + res1 + A4C3N(b,c,d)

% sum of the probabilities of four collisions spread over paths including one to four obstacles

end

function[res] = A4C5N(a,b,c,d,e)

res1 = a^4^ + a^3^ × (b + c + d + e) + a^2^ × (b^2^ + b × (c + d + e) + c^2^ + c × (d + e) + d^2^ + d × e + e^2^);

res2 = a × (b^3^ + b^2^ × (c + d + e) + b × (c^2^ + d^2^ + e^2^ + c × d + c × e + d × e) + c^3^ + c^2^ × (d + e) + c × (d^2^ + e^2^ + d × e) + d^3^ + d^2^ × e + d × e^2^ + e^3^);

res = res2 + res1 + A4C4N(b,c,d,e);

% sum of the probabilities of four collisions spread over paths including one to five obstacles

end

function[res] = A4C6N(a,b,c,d,e,f)

res1 = a^4^ + a^3^ × (b + c + d + e + f) + a^2^ × (b^2^ + b × (c + d + e + f) + c^2^ + c × d + e + f) + d^2^ + d × (e + f) + e^2^ + e × f + e^2^);

res2 = a × (b^3^ + b^2^ × (c + d + e + f) + b × (c^2^ + d^2^ + e^2^ + f^2^ + c × d + c × e + c × f + d × e + d × f + e × f));

res3 = a × (c^3^ + c^2^ × (d + e + f) + c × (d^2^ + e^2^ + f^2^ + d × e + d × f + e × f) + d^3^ + d^2^ × (e + f) + d × (e^2^ + f^2^ + e × f) + e^3^ + e^2^ × f + e × f^2^ + f^3^);

res = res3 + res2 + res1 + A4C5N(b,c,d,e,f);

% sum of the probabilities of four collisions spread over paths including one to six obstacles

end

function[res] = A4C7N(a,b,c,d,e,f,g)

res1 = a^4^ + a^3^ × (b + c + d + e + f + g) + a^2^ × (b^2^ + b × (c + d + e + f + g) + c^2^ + c × (d + e + f + g) + d^2^ + d × (e + f + g) + e^2^ + e × (f + g) + f^2^ + f × g + g^2^);

res2 = a × (b^3^ + b^2^ × (c + d + e + f + g) + b × (c^2^ + d^2^ + e^2^ + f^2^ + g^2^ + c × (d + e + f + g) + d × (e + f + g) + e × (f + g) + f × g));

res3 = a × (c^3^ + c^2^ × (d + e + f + g) + c × (d^2^ + e^2^ + f^2^ + g^2^ + d × (e + d + g) + e × (f + g) + f × g));

res4 = a × (d^3^ + d^2^ × (e + f + g) + d × (e^2^ + f^2^ + g^2^ + e × (f + g) + f × g) + e^3^ + e^2^ × (f + g) + e × (f^2^ + g^2^ + f × g) + f^3^ + f^2^ × g + f × g^2^ + g^3^);

res = res4 + res3 + res2 + res1 + A4C6N(b,c,d,e,f,g);

% sum of the probabilities of four collisions spread over paths including one to seven obstacles

end

function[res] = A4C8N(a,b,c,d,e,f,g,h)

res1 = a^4^ + a^3^ × (b + c + d + e + f + g + h) + a^2^ × (b^2^ + b × (c + d + e + f + g + h) + c^2^ + c × (d + e + f + g + h) + d^2^ + d × (e + f + g + h) + e^2^ + e × (f + g + h) + f^2^ + f × (g + h) + g^2^ + g × h + h^2^);

res2 = a × (b^3^ + b^2^ × (c + d + e + f + g + h) + b × (c^2^ + d^2^ + e^2^ + f^2^ + g^2^ + h^2^ + c × (d + e + f + g + h) + d × (e + f + g + h) + e × (f + g + h) + f × (g + h) + g × h));

res3 = a × (c^3^ + c^2^ × (d + e + f + g+ h) + c × (d^2^ + e^2^ + f^2^ + g^2^ + h^2^ + d × (e + f + g + h) + e × (f + g + h) + f × (g + h) + g × h));

res4 = a × (d^3^ + d^2^ × (e + f + g + h) + d × (e^2^ + f^2^ + g^2^ + h^2^ + e × (f + g + h) + f × (g + h) + g × h) + e^3^ + e^2^ × (f + g + h) + e × (f^2^ + g^2^ + h^2^ + f × (g + h) + g × h) + f^3^ + f^2^ × g + h) + f × (g^2^ + h^2^ + g × h) + g^3^ + g^2^ × h + g × h^2^ + h^3^);

res = res4 + res3 + res2 + res1 + A4C6N(b,c,d,e,f,g);

% sum of the probabilities of four collisions spread over path including one to eight obstacles

end
